# Chimney thoracic endovascular repair for anastomotic leakage after ascending aortic replacement for acute type A aortic dissection: a case report

**DOI:** 10.1186/s44215-023-00031-8

**Published:** 2023-04-12

**Authors:** Ko Sakatsume, Yasushi Kudo, Kazuaki Yanagiya, Masaaki Naganuma, Masaharu Hatakeyama, Shinya Masuda, Koichi Nagaya

**Affiliations:** Department of Cardiovascular Surgery, Aomori Prefectural Central Hospital, 2-1-1 Higashitsukurimichi, Aomori, 030-8553 Japan

**Keywords:** Chimney endovascular technique, TEVAR, Acute type A aortic dissection, Malperfusion, Anastomotic leakage

## Abstract

**Background:**

Anastomotic leakage at the distal anastomotic site after surgery for acute type A aortic dissection (AAD) occasionally leads to malperfusion or false lumen dilatation in the remnant dissected aorta. Open surgery remains the optimal therapy; however, subsequent surgery with re-sternotomy is excessively invasive in elderly and frail patients.

**Case presentation:**

We report the case of an 81-year-old woman who was treated with chimney thoracic endovascular aortic repair (TEVAR) for anastomotic leakage after ascending aortic replacement for AAD. A pressure difference between the upper and lower limbs appeared after the primary surgery, and a computed tomography scan showed a stenotic true lumen in the descending aorta due to a “new tear” at the distal anastomotic site. Chimney TEVAR was performed to direct blood flow into the aortic true lumen in order to restore compromised blood flow to lower body because open surgery is excessively invasive for elderly and frail patients. No endoleak and a better expanded true lumen were detected after the subsequent surgical intervention.

**Conclusions:**

Chimney TEVAR combined with an arch debranching procedure for sealing the new entry at the distal anastomotic site after ascending aortic replacement for AAD may be a viable option for high-risk patients.

## Background

Surgical results for acute type A aortic dissection (AAD) have improved, with a mortality rate of 10 to 15% [1–3]; therefore, the number of patients who require reoperations for residual aortic dissection has increased [4, 5]. Anastomotic leakage at the distal anastomotic site after primary surgery for AAD is one of the reasons for reoperation and occasionally leads to malperfusion or false lumen dilatation in the remnant dissected aorta [6]. Although open repair remains the optimal therapy, subsequent surgery with re-sternotomy is excessively invasive [2] and the risk of reoperation is always a concern in elderly and frail patients [7]. Herein, we report the case of an 81-year-old woman treated with the chimney endovascular technique for anastomotic leakage after ascending aorta replacement for AAD.

## Case presentation

**Fig. 1:**
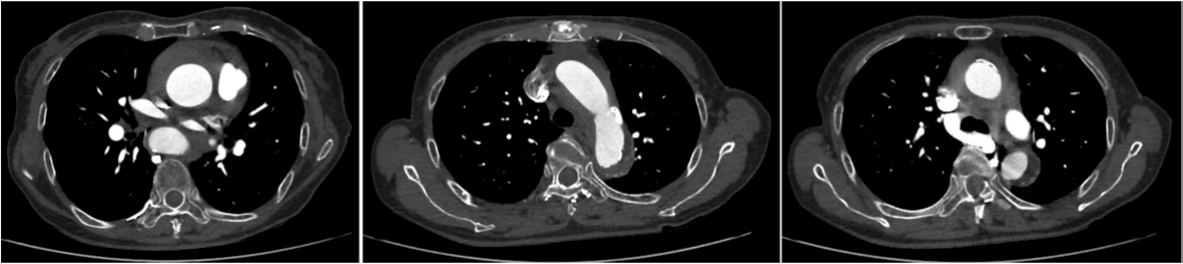
CT findings after emergent transportation. CT shows no primary entry and a thrombosed false lumen from the ascending to descending aorta. CT, computed tomography

**Fig. 2:**
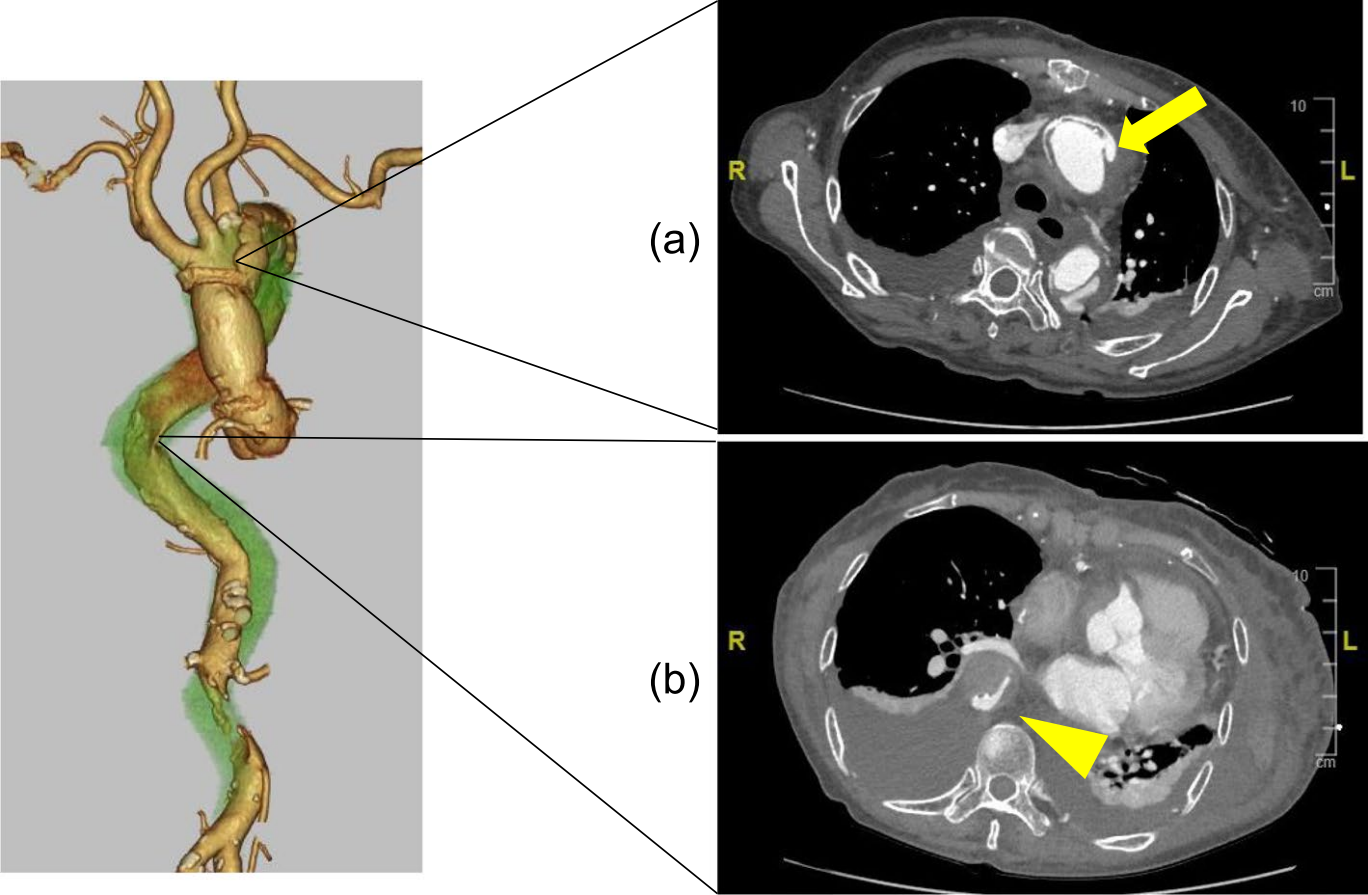
CT findings after the primary surgery. **a** CT shows the new entry (arrow) at the distal anastomotic site. **b** CT indicates stenotic true lumen and expanded false lumen at the descending aorta (arrowhead). CT, computed tomography

An 81-year-old woman with AAD was emergently transferred to our hospital 2 h after the onset of chest and back pain. Computed tomography (CT) showed a Stanford type A aortic dissection extending to the descending aorta without primary entry and with a thrombosed false lumen from the ascending to the descending aorta (Fig. 1). The arch vessels, except for the brachiocephalic artery (BCA), were not dissected. Her vital signs revealed shock due to tamponade; therefore, she was rushed to emergent surgery to save her life. Ascending aorta replacement with a 28-mm Dacron graft was performed. The primary entry from the ascending aorta to the distal aortic arch was not detected during surgery, as shown in the preoperative CT findings (Fig. 1). Her postoperative course was uneventful in the intensive care unit (ICU); however, fatigue in her lower limbs occurred during her rehabilitation program on postoperative day 4, and a pressure disparity of 70 mmHg between her upper and lower limbs was revealed. An emergent CT scan showed a new entry at the distal anastomotic site (Fig. 2a) and a stenotic true lumen from the descending to the abdominal aorta (Fig. 2b). Emergent redo surgery was intended; however, her JAPAN score (Aorta) [8] showed 31.9% mortality and 75.1% morbidity, EuroScore II was 26.45% with specific contribution from renal impairment and surgery on thoracic aorta, and her Clinical Frailty Scale was 5 [9], suggesting that the subsequent surgery with re-sternotomy was an excessively high risk. Therefore, we planned for chimney thoracic endovascular repair (TEVAR) combined with an arch debranching procedure to seal the new tear at the distal anastomotic site and treat the malperfusion in her lower body.

**Fig. 3:**
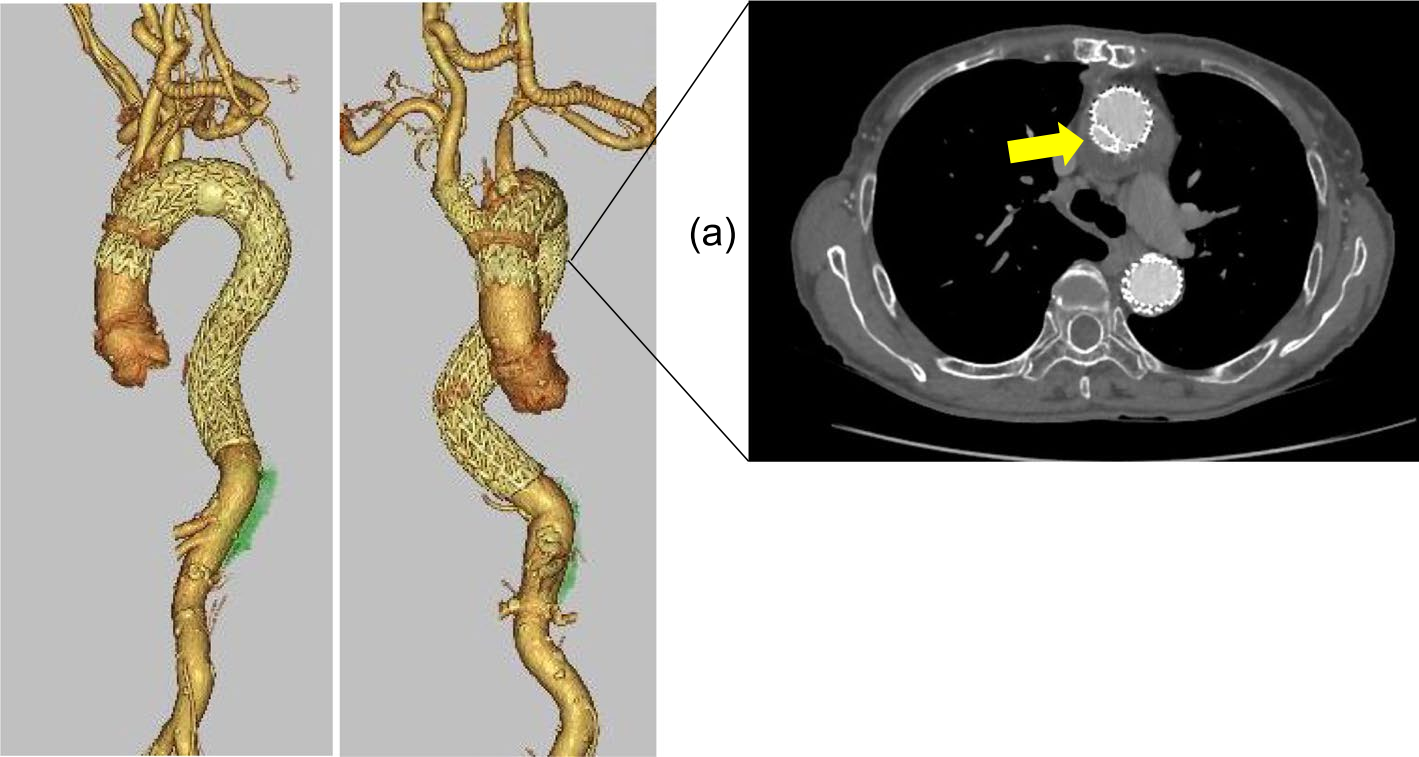
3D-CTA findings after the subsequent surgical intervention. No endoleak is seen, and the true lumen has better expanded below the descending aorta. **a** Axial CT shows a patent Excluder IBE leg at the Chimney part (arrow). 3D-CTA, 3-dimensional computed tomography angiography; CT, computed tomography; IBE, iliac branch endoprosthesis

The arch debranching procedure was initially performed before the chimney TEVAR. Given that the dissection extended to the right subclavian artery (SCA), the right common carotid artery (CCA) was selected for bypass inflow. Right CCA–left CCA–left SCA bypass was performed using the ringed expanded polytetrafluoroethylene (ePTFE) graft, and the proximal site of the left CCA was ligated with no regional saturation of oxygen (rSO_2_) deterioration. The BCA measured 25 mm in length and 12 mm in diameter, and a 28-mm Dacron graft was used for ascending aortic replacement. The plan was to deploy a 16 × 12 × 70 mm Excluder (Gore & Associates, Newark, DE) iliac branch endoprosthesis (IBE) leg from the BCA to the ascending aorta as a chimney graft and a 31 × 31 × 200 mm conformable TAG thoracic endoprosthesis (C-TAG) (Gore & Associates) that was one size larger to prevent gutter endoleaks. A guidewire was inserted through the right common femoral artery (CFA) while checking the true lumen in the aorta under intravascular ultrasound (IVUS) assessment. A Lunderquist (COOK Medical, Bloomington, IN) extra-stiff wire was used to deliver the C-TAG. First, a 28× 28 × 200 mm C-TAG was deployed from the proximal descending aorta to above the celiac artery. Subsequently, the IBE leg was inserted via the right CCA and advanced into the mid-ascending aorta, and then, the planned C-TAG was deployed to adequately overlap the previous stent-graft. Finally, the IBE leg was deployed at the same level as that of the C-TAG device. There were no changes in rSO_2_ during the device deployments. The completed angiogram demonstrated patency of all great vessels, complete exclusion of the false lumen antegrade perfusion in the descending aorta, and no endoleaks. A better expanded true lumen was also assured from the descending aorta to the abdominal aorta using IVUS assessment. The patient recovered well, and the pressure difference between the upper and lower limbs disappeared. Postoperative CT revealed no endoleak and a better-expanded true lumen in the descending aorta (Fig. 3).

## Discussion

Resection of the primary intimal tear and replacement with a graft is an established mainstay treatment for AAD; however, the blood flow from some re-entries to the false lumen is often detected in the distal remnant dissected aorta, accelerating aortic growth [5]. An anastomotic leakage at the distal anastomotic site might be one such re-entry, leading to malperfusion or false lumen dilatation. Furthermore, anastomotic leakage at the anastomotic site is not uncommon. Tanaka et al. [6] reported that 7 of 34 patients (20.5%) who underwent ascending aorta replacement followed by CT scan had distal anastomotic leakage. The number of patients with such complications is expected to increase with improvements in early mortality after immediate surgical repair for AAD.

Our patient underwent ascending aorta replacement because no primary entry was detected from the ascending aorta to the distal aortic arch during primary surgery. Suture cutting at a distal anastomotic site made new major entry result in stenotic true lumen and malperfusion of her lower limbs. A variety of treatment options are available for subsequent surgery. It is first necessary to consider whether surgery with or without re-sternotomy is required from the viewpoint of invasiveness. Redo-open surgery should be performed if the patient is expected to tolerate the risk of surgery since re-sternotomy as open surgery is more curative than one without re-sternotomy. However, the patient was over 80 years old and her expected mortality and morbidity rates were high; thus, we did not select redo-open surgery. The surgery with re-thoracotomy would have been very invasive even if the frozen elephant trunk was used to reduce the invasiveness of the surgery. In addition, Some interventions, such as TEVAR to expand the true lumen of the descending aorta, axillo-bifemoral bypass, and fenestration to the flap were also considered to ameliorate lower body malperfusion. Even though these treatments could contribute to the improvement of blood flow in the acute phase, concerns regarding the expansion of the false lumen in the long term remain because the entry tear is not excluded. Accordingly, we proposed endovascular surgery with entry tear exclusion, a less invasive procedure, although TEVAR with zone 0 landing was required to seal the distal anastomotic site. Moreover, the total arch debranching procedure with re-sternotomy is also highly invasive for elderly and frail women. Hence, we finally proposed to perform a chimney stent-graft technique combined with the two debranching procedures to achieve an adequate seal.

The chimney endovascular technique has emerged as a total endovascular treatment for the management of difficult aortic pathological conditions [10]. This technique utilizes commercially available devices and does not require any device modifications; therefore, this methodology is suitable for urgent additional surgery. Although high clinical success with a low rate of reintervention has been reported [11], gutter-related type 1 endoleaks remain a major issue with the chimney endovascular technique [12]. The combination of the Gore TAG and Medtronic Valiant TEVAR devices with covered stents was reported to be associated with a low rate of gutter endoleaks [11]. In addition, it is recommended that a TEVAR stent-graft that is one size larger than the optimal size and a smaller chimney stent-graft be used to prevent gutter endoleak. Nevertheless, it remains to be seen whether one chimney stent-graft can provide for the entire cerebral blood flow. Hongo et al. reported that the BCA was the preferred source for the entire cerebral blood flow, and a 12-mm or 14-mm stent-graft was placed in the BCA because the BCA had a sufficiently large diameter, being the main trunk downstream of the carotid and subclavian arteries after the arch debranching procedure [13, 14]. C-TAG combined with a 12-mm Excluder IBE leg was used in our procedure; moreover, a large C-TAG was selected to reduce gutter leaks. In addition, the Excluder IBE leg was structurally tapered in the distal segment, which was considered to improve the fitting with the TEVAR stent-graft [15]. All procedures were performed successfully, and no gutter-related type 1 endoleaks were observed on the final angiogram.

## Conclusions

Anastomotic leakage after primary surgery for AAD is not uncommon and occasionally leads to malperfusion or false lumen dilatation in the remnant aortic dissection. A chimney TEVAR combined with an arch debranching procedure for sealing the new entry at the distal anastomotic site may be a viable option for high-risk patients.

## Data Availability

Data will be made available on reasonable request.

## Abbreviations

AAD: Acute type A aortic dissection
TEVAR: Thoracic endovascular aortic repair
CT: Computed tomography
BCA: Brachiocephalic artery
ICU: Intensive care unit
SCA: Subclavian artery
CCA: Common carotid artery
ePTFE: Expanded polytetrafluoroethylene
rSO_2_: Regional saturation of oxygen
IBE: Iliac branch endoprosthesis
C-TAG: Conformable TAG
CFA: Common femoral artery
IVUS: Intravascular ultrasound
3D-CTA: 3-Dimensional computed tomography angiography

## References

[1] Omura A, Miyahara S, Yamanaka K, Sakamoto T, Matsumori M, Okada K, et al. Early and late outcomes of repaired acute DeBakey type I aortic dissection after graft replacement. J Thorac Cardiovasc Surg. 2016;151(2):341–8. 26496808 10.1016/j.jtcvs.2015.03.068

[2] Geirsson A, Bavaria JE, Swarr D, Keane MG, Woo YJ, Szeto WY, et al. Fate of the residual distal and proximal aorta after acute type a dissection repair using a contemporary surgical reconstruction algorithm. Ann Thorac Surg. 2007;84(6):1955–64. 18036916 10.1016/j.athoracsur.2007.07.017

[3] Rylski B, Milewski RK, Bavaria JE, Vallabhajosyula P, Moser W, Szeto WY, et al. Long-term results of aggressive hemiarch replacement in 534 patients with type A aortic dissection. J Thorac Cardiovasc Surg. 2014;148(6):2981–5. 25112930 10.1016/j.jtcvs.2014.05.093

[4] Zierer A, Voeller RK, Hill KE, Kouchoukos NT, Damiano RJ Jr, Moon MR. Aortic enlargement and late reoperation after repair of acute type A aortic dissection. Ann Thorac Surg. 2007;84(2):479–86. 17643619 10.1016/j.athoracsur.2007.03.084

[5] Rylski B, Hahn N, Beyersdorf F, Kondov S, Wolkewitz M, Blanke P, et al. Fate of the dissected aortic arch after ascending replacement in type A aortic dissectiondagger. Eur J Cardiothorac Surg. 2017;51(6):1127–34. 28369453 10.1093/ejcts/ezx062

[6] Tanaka H, Okada K, Kawanishi Y, Matsumori M, Okita Y. Clinical significance of anastomotic leak in ascending aortic replacement for acute aortic dissection. Interact Cardiovasc Thorac Surg. 2009;9(2):209–12. 19454413 10.1510/icvts.2008.201558

[7] Gomibuchi T, Seto T, Komatsu M, Tanaka H, Ichimura H, Yamamoto T, et al. Impact of Frailty on Outcomes in Acute Type A Aortic Dissection. Ann Thorac Surg. 2018;106(5):1349–55. 30086279 10.1016/j.athoracsur.2018.06.055

[8] Shimizu H, Hirahara N, Motomura N, Miyata H, Takamoto S. Status of cardiovascular surgery in Japan between 2017 and 2018: A report based on the Japan Cardiovascular Surgery Database. 4. Thoracic aortic surgery. Asian Cardiovasc Thorac Ann. 2021;29(4):278–88. 33342246 10.1177/0218492320981456

[9] Rockwood K, Song X, MacKnight C, Bergman H, Hogan DB, McDowell I, et al. A global clinical measure of fitness and frailty in elderly people. CMAJ. 2005;173(5):489–95. 16129869 10.1503/cmaj.050051 PMC1188185

[10] Ohrlander T, Sonesson B, Ivancev K, Resch T, Dias N, Malina M. The chimney graft: a technique for preserving or rescuing aortic branch vessels in stent-graft sealing zones. J Endovasc Ther. 2008;15(4):427–32. 18729550 10.1583/07-2315.1

[11] Bosiers MJ, Donas KP, Mangialardi N, Torsello G, Riambau V, Criado FJ, et al. European Multicenter Registry for the Performance of the Chimney/Snorkel Technique in the Treatment of Aortic Arch Pathologic Conditions. Ann Thorac Surg. 2016;101(6):2224–30. 26794885 10.1016/j.athoracsur.2015.10.112

[12] Kanaoka Y, Ohki T, Maeda K, Shukuzawa K, Baba T, Tezuka M, et al. Outcomes of Chimney Thoracic Endovascular Aortic Repair for an Aortic Arch Aneurysm. Ann Vasc Surg. 2020;66:212–9. 30802578 10.1016/j.avsg.2018.12.087

[13] Hongo N, Miyamoto S, Shuto R, Wada T, Matsumoto S, Kiyosue H, et al. Endovascular aortic arch reconstruction using in situ stent-graft fenestration in the brachiocephalic artery. J Vasc Interv Radiol. 2011;22(8):1144–8. 21801994 10.1016/j.jvir.2011.04.002

[14] Hongo N, Miyamoto S, Shuto R, Wada T, Kamei N, Sato A, et al. "Squid-capture" modified in situ stent-graft fenestration technique for aortic arch aneurysm repair. Cardiovasc Intervent Radiol. 2014;37(4):1093–8. 24943916 10.1007/s00270-014-0933-y

[15] DeRoo E, Harris D, Olson S, Panthofer A, Meadows W, Pauli T, et al. Conformability of the GORE EXCLUDER iliac branch endoprosthesis is associated with freedom from adverse iliac events. J Vasc Surg. 2021;74(5):1558–64 e1. 34082005 10.1016/j.jvs.2021.05.026

